# MDFN: Enhancing Power Grid Image Quality Assessment via Multi-Dimension Distortion Feature

**DOI:** 10.3390/s25113414

**Published:** 2025-05-29

**Authors:** Zhenyu Chen, Jianguang Du, Jiwei Li, Hongwei Lv

**Affiliations:** Big Data Center, State Grid Corporation of China, Beijing 100031, China; jianguangdu@outlook.com (J.D.); lijiwei0902@163.com (J.L.);

**Keywords:** power grid, image quality assessment, multi-dimension distortion features, frequency selection, brightness, noise

## Abstract

Low-quality power grid image data can greatly affect the effect of deep learning in the power industry. Therefore, adopting accurate image quality assessment techniques is essential for screening high-quality power grid images. Although current blind image quality assessment (BIQA) methods have made some progress, they usually use only one type of feature and ignore other factors that affect the quality of images, such as noise and brightness, which are highly relevant to low-quality power grid images with noise, underexposure, and overexposure. Therefore, we propose a multi-dimension distortion feature network (MDFN) based on CNN and Transformer, which considers high-frequency (edges and details) and low-frequency (semantic and structural) features of images, along with noise and brightness features, to achieve more accurate quality assessment. Specifically, the network employs a dual-branch feature extractor, where the CNN branch captures local distortion features and the Transformer branch integrates both local and global features. We argue that separating low-frequency and high-frequency components enables richer distortion features. Thus, we propose a frequency selection module (FSM) which extracts high-frequency and low-frequency features and updates these features to achieve global spatial information fusion. Additionally, previous methods only use the CLS token for predicting the quality score of the image. Considering the issues of severe noise and exposure in power grid images, we design an effective way to extract noise and brightness features and combine them with the CLS token for the prediction. The results of the experiments indicate that our method surpasses existing approaches across three public datasets and a power grid image dataset, which shows the superiority of our proposed method.

## 1. Introduction

The quality of power grid image samples is crucial for the success of AI solutions within the power sector. In this field, images are widely used for the identification [1], detection [2], analysis, and monitoring of power grid equipment and systems [3]. However, these images are often affected by quality issues such as noise, blurriness, and underexposure or overexposure. Image quality assessment (IQA) methods aim to assess the quality of images based on subjective human perception. According to whether an original reference image is required or not, IQA is usually categorized into full-reference IQA [4], reduced-reference IQA [5], and blind IQA [6]. The scope of the FR-IQA and RR-IQA methods is limited because obtaining the original reference images is challenging in real-world applications. In contrast, BIQA methods do not require other images for reference and thus have greater application potential, but also face higher technical challenges.

Currently, BIQA methods are classified into convolutional neural network (CNN)-based methods [7,8] and Vision Transformer (ViT)-based methods [9]. HyperIQA [8] introduces a module that focuses on local distortions to capture local features, while a hyper-network is utilized to generate the parameters for the network. DEIQT [10] uses the Transformer architecture in the encoder-decoder structure and feeds CLS tokens [9] into the decoder, and finally outputs image quality scores. LoDa [11] combines the advantages of CNN and Transformer by embedding local distortion features extracted by CNN into a pretrained ViT model and predicting image quality scores by CLS tokens.

**Figure 1:**
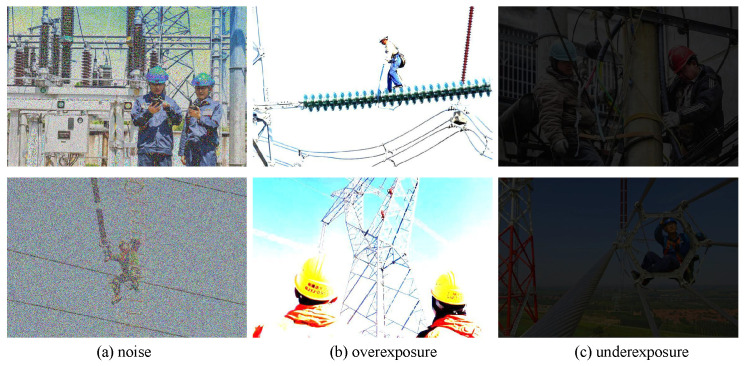
Distortion images from power grid dataset.

As illustrated in Figure 1, power grid images often experience significant distortions such as noise, overexposure, or underexposure. Previous methods [8,10,11] have generally overlooked these specific characteristics, resulting in a decrease in quality prediction accuracy for these images. Noise typically corresponds to high-frequency features, while exposure levels are related to image brightness. We argue that integrating these features into image quality assessment models can enhance prediction accuracy. Meanwhile, low-frequency features, which contain semantic information and various local distortion characteristics, are also essential for predicting the image quality. In addition, CNNs are effective at extracting local texture features, while ViTs can integrate global features of the image. Combining the strengths of both is crucial for improving image quality predictions in power grid applications.

**Figure 2:**
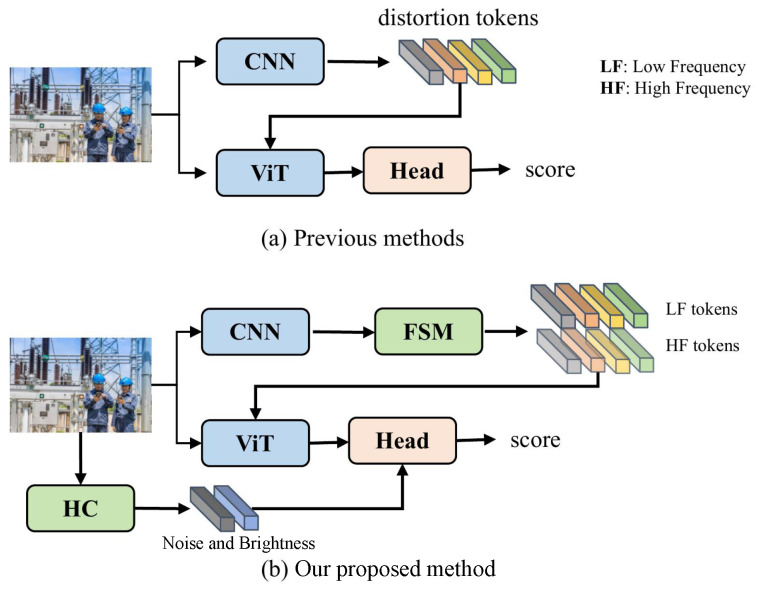
The difference between previous methods and our network. FSM denotes the frequency selection module and HC denotes the extraction of the brightness and noise features.

Finally, a multi-dimension distortion feature network (MDFN) is proposed based on CNN and Transformer, which leverages low-frequency features, high-frequency features, noise, and brightness information to assess the quality. Specifically, we design a dual-branch feature extractor, where CNN extracts multi-scale local distortion features, while ViT captures global image features, with extracted distortion features from CNN additionally introduced at each layer. As shown in Figure 2, previous methods typically do not separate low-frequency and high-frequency features when extracting distortion features. We argue that more detailed distortion features can be obtained by processing low-frequency and high-frequency features separately, since high frequencies are typically associated with edges and details, while low frequencies are generally related to the semantic features. Therefore, we propose the frequency selection module (FSM) to extract low-frequency and high-frequency features, and update these representations in the spectral domain to achieve feature fusion in the spatial domain. Specifically, it first converts the features into the spectral domain using an FFT and passes them through a filter to get the frequency features, and then combines the real and imaginary components of the representations along the channel dimension to obtain the spectral domain representations. Then, the features are calculated with convolutional layers to realize the update of the frequency features. This can be regarded as a global fusion of the features because a change in the value within the spectral domain will influence all values in the spatial field. Finally, the real and imaginary parts are separated and the features are converted back to the spatial field to obtain multi-scale low-frequency and high-frequency distortion features. Then, inspired by LoDa [11], we introduce a local distortion feature injection module that incorporates local distortion features into ViT. Finally, the noise and brightness features of the image are hand-designed and concatenated with the CLS token from ViT to perform the final quality prediction. As illustrated in Figure 2, previous methods ignore the importance of the noise and brightness features and do not combine them into the prediction of image quality. Therefore, we design an effective method to obtain these features. Specifically, the brightness features can be viewed as an average of the gray values of the RGB channels of the image. For noise features, Gaussian blurring is first applied to the image, then the residuals of the image before and after blurring are calculated, and we finally obtain the standard deviation of the residuals. Considering the number of parameters, average pooling is finally performed on the hand-designed features.

The results of the experiments indicate that our approach surpasses current models on three public datasets, with particularly significant improvements observed on the power grid image dataset, which demonstrates the success of our method. The main contributions of the paper are as follows:

- The frequency selection module (FSM) is proposed to split the distortion features into the low-frequency and high-frequency representations and obtain the more detailed distortion tokens.
- Considering the traits of the power grid image, we suggest extracting the brightness and noise features and combining them with the CLS token for better quality prediction.
- We propose the multi-dimension distortion feature network (MDFN) based on CNN and Transformer. It leverages low-frequency features, high-frequency features, noise, and brightness features and can achieve more accurate predictions.

## 2. Related Work

### 2.1. Deep Learning-Based Image Quality Assessment

The outstanding performance demonstrated by deep learning across a range of applications on computer vision [12,13] has driven its wide application in image quality assessment (IQA). Currently, the research methods in this field are mainly categorized into convolutional neural network (CNN)-based and Transformer-based. CNN methods usually obtain low-level spatial representations of an image from the initial layer of the network, while more complex high-level semantic information is captured in subsequent layers. MRLIQ [13] proposes a framework for multi-task learning that incorporates subjective quality scores and distortion types during training to improve IQA performance. DeepBIQ [14] estimates the overall image quality by scoring multiple sub-regions divided by the original image and pooling these scores, with the score of each sub-region being determined by a Support Vector Machine (SVM) computation. Some methods [15,16] explore the possibility of introducing a reference image to improve quality prediction performance in a reference-free environment. Hallucinated-IQA [17], in contrast, employs a generative adversarial network to create synthetic reference images to address the absence of real reference images, and integrates the distorted images for quality score prediction. NIMA [18] proposes a CNN-based model that estimates the perceived range of quality scores, rather than predicting the average score. The meta-learning model [19] focuses on learning a shared prior across multiple distortion types to strengthen the ability of the IQA network. HyperIQA [8], on the other hand, achieves efficient image quality prediction through the multi-scale extraction and fusion of the features.

In traditional IQA methods, CNNs are typically employed as the primary backbone network for the extraction of features. Although CNNs are good at capturing the local feature structure of an image, their inherent localization bias makes them face certain limitations in modeling non-local dependencies. In addition, the property of CNNs to share weights at all spatial locations introduces spatial invariance, a mechanism that somewhat limits their ability to model complex feature interactions. Transformer architecture [20] was originally designed for NLP tasks, but has shown great potential in CV in recent years. Vision Transformer (ViT) [9] is a prominent model in the field, realizing image classification tasks with a pure Transformer encoder and gradually catching up with CNN-based approaches in terms of performance. Transformer has significant advantages in modeling non-local dependencies of images, making it a potential method in IQA tasks.

Given that the IQA task needs to capture both local and global feature connections, some studies have attempted to combine Transformer with CNN to fully utilize the advantages of both. For example, TRIQ [21] uses Transformer to further process CNN-extracted features for quality prediction. TReS [22] feeds multiscale features into Transformer to model non-local information, which ultimately outputs the quality scores. DEIQT [10] uses the Transformer decoder to enhance the CLS token representation of the encoder to optimize the quality prediction performance from multiple perspectives. AHIQ [23] introduces a hybrid network based on an attention mechanism that merges the global features captured by ViT with the local texture details obtained through CNN. MANIQA [24] significantly improves the performance through the multi-dimensional attention mechanism, which achieves the co-optimization of local and global information across both the channel and spatial domains.

### 2.2. Frequency-Domain Learning

Recently, frequency-domain techniques have been increasingly incorporated into deep learning. Some studies [25,26,27] have explored the optimization mechanism of deep neural networks (DNNs) and their generalization ability. Rahaman et al. [28] pointed out that during the training process, the objective function of a deep network tends to preferentially fit low-frequency components. AdaBlur was proposed [29] to alleviate the aliasing problem by employing a content-aware low-pass filter in the downsampling process. Meanwhile, FLC [30] proved that frequency aliasing may weaken the robustness of the model. In addition, frequency-domain methods have also been used to enhance the attention mechanism. Qin et al. [31] has proposed to utilize the frequency information obtained from the DCT as an additional feature of the channel attention mechanism. Huang et al. [32], on the other hand, applied the classical convolution theorem in a deep network and demonstrated that an adaptive frequency filter can act as a global token mixer, greatly enhancing the capability of the network to capture features. In addition, many studies [33,34] have attempted to integrate frequency-domain methods into deep network structures to enhance the learning ability of non-local features.

## 3. Methodology

### 3.1. Overall Architecture

**Figure 3:**
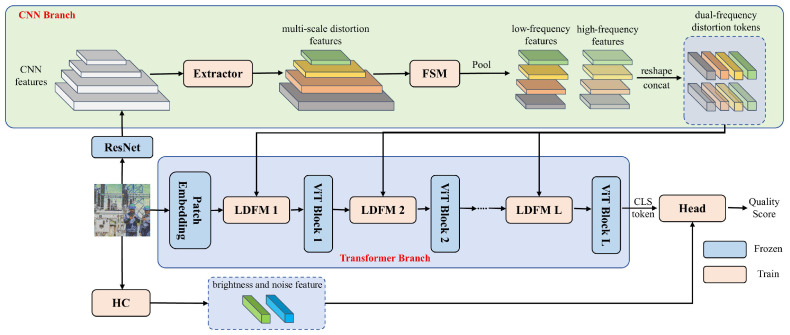
Framework overview of the proposed MDFN. There are two branches, where the CNN branch extracts the distortion features and the Transformer branch fuses the distortion features for quality prediction. Extractor, FSM, LDFM, and HC denote the distortion extractor, frequency selection module, local distortion fusion module, and hand-crafted, respectively.

Our MDFN employs a dual-branch architecture, where the CNN branch is dedicated to capturing distortion features, while the Transformer branch utilizes a pretrained ViT to enhance the extraction of quality-related information. As shown in Figure 3, for an image $I\in R^{3\times H\times W}$, the CNN branch firstly applies ResNet50 to get image features of different sizes. These features are subsequently processed through an Extractor to obtain multi-scale distortion features $f\;=\;\{f_{i}\in R^{C\times \frac{H}{2^{i+1}}\times \frac{W}{2^{i+1}}}|i\;=\;1,2,3,4\}$, where *C* represents the dimension of the channel, and *H* and *W* indicate the height and width. We believe that separating the low-frequency and high-frequency of these features can allow for better capture of detailed distortion information. Therefore, a feature selection module (FSM) is proposed to extract both components. Simultaneously, a pooling layer is employed for downsampling, resulting in low-frequency features $f_{l}=\left\{f_{l}^{i}\in R^{C\times \frac{H}{32}\times \frac{W}{32}}|i\;=\;1,2,3,4\right\}$ and high-frequency features $f_{h}=\left\{f_{h}^{i}\in R^{C\times \frac{H}{32}\times \frac{W}{32}}|i=1,2,3,4\right\}$. We then apply a reshape operation $R^{C\times H\times W}\to R^{C\times HW}$ to process the features, and then concatenate them along the last dimension to form the final dual-domain distortion tokens $F_{d}\in R^{8N\times C}$, where $N=\frac{H}{32}\times \frac{W}{32}$.

In the Transformer branch, we freeze the weights and insert a local distortion fusion module between each layer to integrate the distortion tokens extracted by the CNN branch, which helps the pretrained ViT network capture richer distortion features and obtain more accurate quality scores. To address the specific characteristics of the power grid images, we design brightness and noise features, which are concatenated with the CLS token output by the ViT and then passed into a regression layer to predict the quality score.

Note that only the extractor, local distortion fusion module, feature selection module, and regression head are trainable, while the parameters of the pretrained ViT and CNN remain frozen.

### 3.2. Frequency Selection Module

Previous studies [26,35] on the spectrum domain and deep learning have indicated that high-frequency components correspond to areas with rapid pixel changes, such as edges, textures, and noise, while low-frequency components capture broader semantic details. Traditional image quality assessment methods have rarely extracted distortion features from the perspective of frequency. In fact, processing low-frequency and high-frequency components separately can yield richer distortion features. Additionally, compared to high-quality images, low-quality images often exhibit different frequency distributions. For example, images with significant noise tend to have more high-frequency components. Extracting these frequency features for image quality assessment can improve prediction accuracy. However, it is insufficient to extract only low-frequency or high-frequency features. Integrating these features with the overall image features can achieve comprehensive feature fusion. According to Fourier theory, altering a single frequency value affects the entire data globally. This principle motivates the use of non-local receptive fields for design operations. Therefore, processing the features from the frequency domain can enable global feature fusion in the spatial domain.

**Figure 4:**
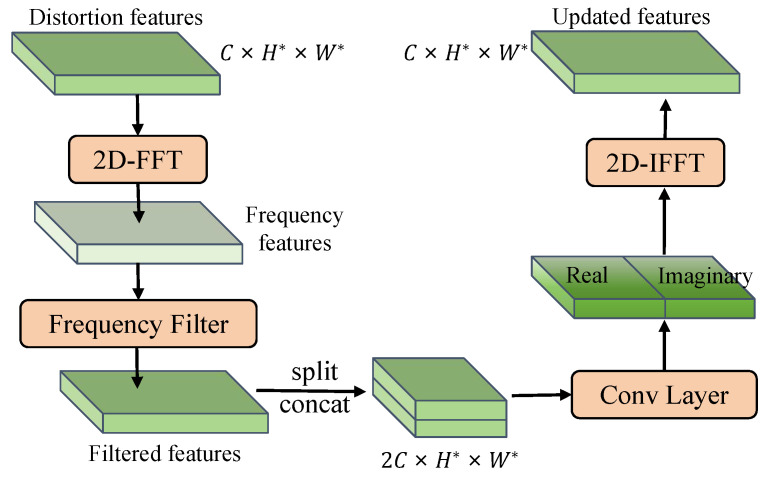
The architecture of the frequency selection module (FSM).

Specifically, the frequency selection module (FSM) is proposed, which separates the features into low- and high-frequency components and updates them individually in the frequency domain, allowing for the extraction of finer distortion details. As shown in Figure 4, the distortion features *F* are first converted into the frequency domain through a 2D FFT. Then, a frequency filter (e.g., low-pass or high-pass) is applied to obtain the filtered features $F_{filter}$, which emphasizes either low or high frequencies. The formula is as follows:

(1) $$\begin{array}{cc} F_{freq}\left(u,v\right) & =\sum_{x=0}^{m-1}\sum_{y=0}^{n-1}F\left(x,y\right)\cdot e^{-j2\pi \left(\frac{ux}{m}+\frac{vy}{n}\right)}, \end{array}$$

(2) $$\begin{array}{cc} F_{filter} & =Filter(F_{freq}) \end{array}$$

where Filter denotes the filter, $F_{freq}$ is the frequency domain feature.

Since frequency features typically consist of real and imaginary parts, both of which are crucial for feature learning, we separate these two components and concatenate them on the channel dimension to form the concatenated features $F_{concat}\in R^{2C\times H^{*}\times W^{*}}$. Next, these concatenated features are passed through a convolutional layer, which includes a 1×1 convolution and an activation function, to update the frequency features. The updated features $F_{update}$ are then split along the channel dimension and converted back to frequency components. Afterwards, we perform a 2D-IFFT to transform the features $F_{update}$ to the spatial domain. Finally, the transformed features $F_{\mathrm{spatial}}$ are combined with the initial distortion representations to yield the updated distortion features $F_{final}$. The formula is as follows:

(3) $$\begin{array}{cc} F_{spatial}\left(x,y\right) & =\frac{1}{mn}\sum_{u=0}^{m-1}\sum_{v=0}^{n-1}F_{\mathrm{update}}\left(u,v\right)\cdot e^{j2\pi \left(\frac{ux}{m}+\frac{vy}{n}\right)}, \end{array}$$

(4) $$\begin{array}{cc} F_{final} & =F+F_{spatial} \end{array}$$

### 3.3. Distortion Extractor

The features extracted by CNNs may not necessarily align with the distortion features required for the quality assessment of images. To address this, we design a distortion extractor to further capture distortion-specific features and adjust their channel dimensions for consistent processing. Moreover, relying solely on the features from the last layer is insufficient, as they primarily represent the overall image content and lack detailed information. To obtain richer distortion features, we utilize multi-scale features for extraction.

**Figure 5:**
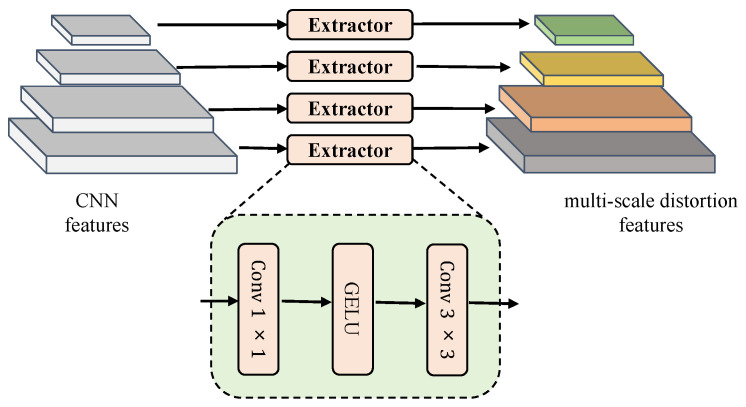
The architecture of the distortion extractor.

Specifically, as illustrated in Figure 5, we employ four different network branches to extract degradation features at each scale. Feature $F_{i}$ is processed sequentially through a 1×1 convolution, a GELU activation function [36], and a 3×3 convolution to produce the distortion features. The formula is as follows:

(5) $$f_{i}=Conv_{3\times 3}\left(GELU\left(Conv_{1\times 1}\left(F_{i}\right)\right)\right)$$

where $f_{i}\in R^{C\times \frac{H}{2^{i+1}}\times \frac{W}{2^{i+1}}}$ denotes the output feature. Next, the obtained distortion features are input into the frequency selection module.

### 3.4. Hand-Crafted Features and IQA Regression

In image processing and computer vision, brightness and noise are two key low-level features that directly affect image visibility and stability. As power grid images are often noisy and exhibit significant brightness features, we design a method for extracting brightness and noise features, which are combined with the CLS token output from the ViT model, allowing for the effective utilization of fundamental image information in image quality assessment.

Brightness features reflect the overall illumination intensity of an image and can effectively describe both local and global light distribution. For each image $I\in R^{3\times H\times W}$, the brightness features are computed by taking the average of the RGB channels to minimize the impact of color bias on brightness estimation. The calculation process for the brightness features is as follows:

(6) $$F_{bright}=\frac{1}{3}\sum_{c=1}^{3}I_{c}$$

where $F_{bright}\in R^{1\times H\times W}$ is the brightness representation of the single channel and $I_{c}$ denotes the *c*-th channel of the image.

Noise features reflect the distribution of random interference information in the image and are important indicators of image quality. Gaussian blur is a low-pass filter that removes high-frequency representations while preserving the low-frequency representations of an image. Therefore, an image after Gaussian blur can be considered the low-frequency part of the original image. Since the original image contains all frequencies, a residual image with the high-frequency components can be obtained by subtracting the Gaussian blurred image from the original image. Specifically, a Gaussian blur operation is employed on the original image to obtain a blurred image $I_{blur}$, with a 5×5 blur kernel to smooth the details of the image. The equation is as follows:

(7) $$\begin{array}{cc} I_{\mathrm{blur}} & =I_{c}*G, \end{array}$$

We then calculate the residual between the blurred image and the original image and compute the root mean square value of the residual in local regions to estimate the noise intensity. The formula is as follows:

(8) $$F_{noise}=\sqrt{\frac{1}{3}\sum_{c=1}^{3}{\left(I_{c}-I_{blur,c}\right)}^{2}}$$

where $F_{noise}\in R^{1\times H\times W}$ is the noise representation of the single channel.

To reduce computational complexity, the brightness and noise features are downsampled to a resolution of $\frac{H}{32}\times \frac{W}{32}$ with adaptive average pooling, and are concatenated across the channel dimension to yield the hand-crafted features $F_{hc}\in R^{2\times \frac{H}{32}\times \frac{W}{32}}$. The formula is as follows:

(9) $$F_{hc}=Concat\left(F_{bright},F_{noise}\right)$$

With the output CLS token $F_{c}\in R^{1\times D}$ and the hand-crafted features $F_{hc}\in R^{2\times \frac{H}{32}\times \frac{W}{32}}$, we first flatten the features into a one-dimensional vector and concatenate them to obtain the features $F_{q}\in R^{L}$ for quality prediction, where $L=D+2\times \frac{H}{32}\times \frac{W}{32}$. Finally, the features are fed into a single-layer regressor head to obtain the quality score. We use the PLCC-induced loss for training. Given *m* images on the training batch and the predicted quality scores $\hat{y}=\left\{{\hat{y}}_{1},{\hat{y}}_{2},...,{\hat{y}}_{m}\right\}$ and corresponding label $y=\{y_{1},y_{2},...,y_{m}\}$, the loss is calculated as:

(10) $$L_{plcc}=\left(1-\frac{\sum_{i=1}^{m}\left({\hat{y}}_{i}-\hat{a}\right)\left(y_{i}-\hat{a}\right)}{\sqrt{\sum_{i=1}^{m}{\left({\hat{y}}_{i}-\hat{a}\right)}^{2}\sum_{i=1}^{m}{\left(y_{i}-\hat{a}\right)}^{2}}}\right)/2$$

### 3.5. Local Distortion Fusion Module

A straightforward approach to fusing dual-frequency distortion tokens into a pretrained ViT model involves simply adding the features to the tokens. However, image tokens of ViT correspond to 16×16 patches of the original image, which may not align with the scale of the dual-frequency distortion features. To address this misalignment, inspired by LoDa [11], we introduce the cross-attention mechanism, which enables the ViT image tokens to query similar features from the dual-frequency distortion features. The queried features are then effectively integrated with the image tokens, which ensures the coherent and effective incorporation of distortion information.

**Figure 6:**
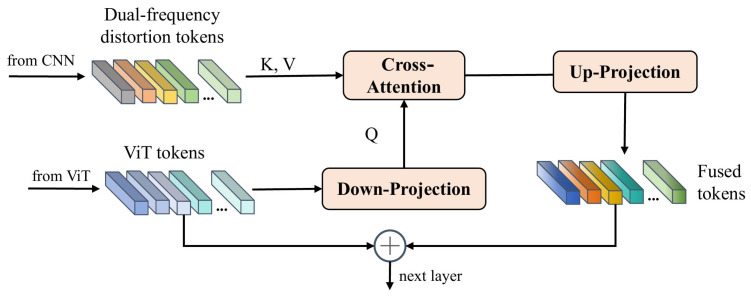
The architecture of the local distortion fusion module (LDFM).

Considering that the channel dimension of ViT features is 768, while the channel dimension of the dual-domain token is 256, we need to ensure channel consistency. To achieve this, we first process the channel dimensions. As shown in Figure 6, the input tokens from the ViT are passed through a down-projection layer to map channel dimensions to match that of the dual-domain distortion tokens. The formula is as follows:

(11) $$F_{vit}^{\prime }=Down\left(F_{vit}\right)$$

where Down is the down-projection layer, which is a trainable MLP layer and performs the projection of ViT token $F_{vit}\in R^{M\times D}$ into $F_{vit}^{\prime }\in R^{M\times C}$.

Then, the cross-attention network is applied for the fusion between the ViT tokens and the dual-frequency distortion tokens. Specifically, the processed ViT tokens $F_{vit}^{\prime }$ are considered as query *Q*, and the dual-frequency distortion tokens are viewed as key *K* and value *V* in the multi-head cross-attention (MHCA). The equation is as follows:

(12) $$\begin{array}{cc} F_{cross} & =MHCA(Q,K,V)+Q, \end{array}$$

(13) $$\begin{array}{cc} MHCA(Q,K,V) & =softmax(\frac{Q\cdot K}{\sqrt{d}})\cdot V, \end{array}$$

where the softmax function transforms a vector $z=[z_{1},z_{2},\cdots ,z_{n}]$ into a probability distribution, and is given by the following formula:

(14) $$\mathrm{softmax}\left(z_{i}\right)=\frac{e^{z_{i}}}{\sum_{j=1}^{n}e^{z_{j}}},$$

where $z_{i}$ is the *i*-th value of the vector z, *n* is the dimension of z, and $e^{z_{i}}$ is the exponential of the input value. After that, the fused tokens $F_{cross}$ are added with the initial ViT tokens $F_{vit}$ to keep the initial features, which are expressed as follows:

(15) $$F_{vit}^{\prime }=F_{cross}+F_{vit}^{\prime }$$

Finally, an up-projection layer is used to transform the dimension of the fused features back to a format compatible with the ViT model. Note that the up-projection layer is essentially also an MLP.

## 4. Experiments

### 4.1. Used Datasets and Metrics for Evaluation

#### 4.1.1. Used Datasets

In this experiment, three publicly available datasets are used: LIVE [37], LIVEC [38], and TID2013 [39]. In addition, a private power grid dataset is used to assess the effectiveness of the method on the power grid images. There are 779 synthetically distorted images in the LIVE dataset. In addition, it contains five types of distortions. TID2013 includes 3000 synthetically distorted images, featuring 24 different types of distortions. LIVEC includes 1162 images from mobile devices, which exhibit varying degrees of authentic distortions. The power grid dataset contains 1110 images with different distortions, including noise, blur, overexposure, underexposure, and tilt.

#### 4.1.2. Metrics for Evaluation

The experimental results are evaluated with two common metrics: the Pearson linear correlation coefficient (PLCC) and Spearman’s rank-order correlation coefficient (SRCC). PLCC is a measure of the linear relationship between two variables, with values ranging from −1 to +1. It is primarily employed to assess the linear correlation between estimated values and true values. SRCC evaluates the consistency of the rankings between two variables, rather than just their numerical relationship. Unlike PLCC, SRCC does not require a linear relationship between the variables and focuses on the order of the rankings.

### 4.2. Implementation Details

All experiments are performed on an 11GB GPU. The model is built and trained using the PyTorch 1.10 framework. The AdamW optimizer is utilized, the learning rate is 0.0001, and the weight decay is 0.01. A cosine learning rate schedule is employed to change the learning rate dynamically during training. For each dataset, training is conducted over 10 runs, with random splits for the training and validation sets in each run. Each experiment runs for 30 epochs. Evaluation is carried out using the PLCC and SRCC metrics.

We use the ResNet50 and ViT-Base pretrained on the ImageNet-21K as the backbone of the CNN branch and Transformer branch, respectively. The input image is cropped to 224×224.

### 4.3. Experiment Results on LIVE Dataset

Table 1 presents the comparison of the proposed MDFN and other methods on the LIVE dataset. The proposed MDFN surpasses all existing methods on the LIVE dataset, achieving SRCC and PLCC scores of 0.981 and 0.983, respectively. Specifically, compared to LoDa, our method surpasses it by 0.006 in SRCC and 0.004 in PLCC. LoDa [11] is our baseline method but it does not incorporate frequency, noise, or brightness features, which results in lower performance compared to our method. This demonstrates the benefit of integrating these features for quality prediction. Furthermore, compared to the Transformer-based method MUSIQ [40], our results show a substantial improvement, surpassing MUSIQ by 0.041 in SRCC and by 0.072 in PLCC, which highlights that incorporating CNN-extracted distortion features into ViT significantly enhances prediction accuracy.

### 4.4. Experiment Results on LIVEC Dataset

As illustrated in Table 2, our MDFN also outperforms existing methods on the more challenging LIVEC dataset. Specifically, our method achieves SRCC and PLCC scores of 0.889 and 0.903, respectively. In terms of SRCC, our approach surpasses LoDa by 0.013 and DEIQT by 0.014. DEIQT uses an initialized query, which is fed into the transformer decoder for quality prediction, but does not leverage frequency features, resulting in limited distortion feature learning. In contrast, our proposed frequency selection module (FSM) further decomposes features into low-frequency and high-frequency components, capturing more comprehensive distortion features. For the PLCC metric, our method exceeds LoDa [11] and DEIQT [10] by 0.004 and 0.009, respectively. Compared to MUSIQ [40], our approach shows a substantial improvement, outperforming it by 0.157 in PLCC. These results indicate that our method can achieve excellent performance even in more complex scenarios.

### 4.5. Experiment Results on TID2013 Dataset

Compared to the LIVE and LIVEC datasets, the TID2013 dataset presents a greater variety of degradation types and more complex scenarios. As illustrated in Table 3, our method obtains SRCC and PLCC scores of 0.880 and 0.909, respectively. Compared with the baseline method LoDa, our approach demonstrates a clear improvement, surpassing it by 0.011 in SRCC and 0.008 in PLCC. This validates that integrating multiple distortion features can effectively enhance quality prediction accuracy. Additionally, compared with TReS, our method outperforms it by 0.017 in SRCC and 0.026 in PLCC. Although TReS combines CNN and Transformer architectures, it still relies solely on multi-scale features to generate distortion features, which limits the learning of the distortion features. This further demonstrates the effectiveness of low-frequency and high-frequency distortion features for quality prediction.

### 4.6. Experiment Results on Power Grid Dataset

We trained other methods on this dataset and compared their performance with our proposed MDFN. As illustrated in Table 4, our method surpasses the other methods on all metrics. Compared to the CNN-based method HyperIQA, our approach surpasses it by 0.012 in PLCC and 0.008 in SRCC. Compared to Transformer-based methods, our method outperforms TIQA by 0.014 in PLCC and 0.01 in SRCC, and DEIQT by 0.015 and 0.01 in PLCC and SRCC, respectively. For the baseline method LoDa, our approach obtains gains of 0.007 and 0.005 on the PLCC and SRCC, respectively. These results indicate that our method also has a clear merit on the power grid dataset, further demonstrating its effectiveness.

### 4.7. Ablation Study

#### 4.7.1. Component Analysis

There are three main components in our network: the local distortion fusion module (LDFM), the feature selection module (FSM), and hand-crafted distortion features (HC). To examine the role of each module, experiments of ablation are performed, as illustrated in Table 5. It can be observed that omitting FSM or HC leads to a certain performance drop. If we only use the hand-crafted distortion features and do not use features from CNN, it will cause a significant drop in performance, which highlights the importance of integrating CNN and ViT features. Combining all features yields the best performance, demonstrating that merging multiple distortion features is a reasonable approach that enables a more comprehensive understanding of the image.

#### 4.7.2. Performance with Different CNN Backbones

As shown in Table 6, we evaluated the performance of our network with different CNN backbones on the power grid dataset, specifically ResNet18 [47], VGG16 [48], and ResNet50. The results indicate that ResNet50 achieves the highest performance, with a PLCC of 0.989 and an SRCC of 0.981, outperforming both ResNet18 and VGG16. Using ResNet18 as the backbone yields PLCC and SRCC scores of 0.978 and 0.974, respectively, while VGG16 slightly improves these scores to 0.979 for PLCC and 0.975 for SRCC. ResNet50 outperforms them in both metrics, which suggests that deeper architectures are more effective at capturing distortion features relevant to image quality in power grid images. The improved performance with ResNet50 may be attributed to its ability to extract richer feature representations, which improves the overall quality assessment accuracy.

#### 4.7.3. Performance with Different Frequency Features

Table 7 presents an evaluation of the performance of the power grid dataset with different frequency components: low-frequency, high-frequency, and a combination of both. When only low-frequency features are used, the model obtains a PLCC of 0.979 and an SRCC of 0.975. Using only high-frequency features slightly improves the results, with a PLCC of 0.981 and an SRCC of 0.976. However, the best performance is generated with employing both low-frequency and high-frequency features, yielding a PLCC of 0.989 and a notably higher SRCC of 0.981. This improvement suggests that both frequency components contribute complementary information for image quality assessment, with low-frequency components capturing global structure and brightness and high-frequency components emphasizing fine details and edges.

**Figure 7:**
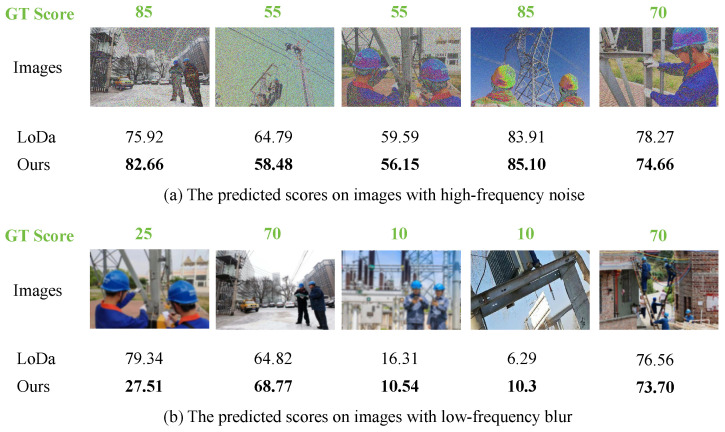
Comparison of the predicted score between the baseline method LoDa and our method MDFN.

In addition, to further show the superiority of our method on images with different frequency components, the estimated scores from the baseline and our method are shown. As shown in Figure 7, when the image contains a lot of noise, which means that it has more high-frequency components, our method predicts image quality more accurately than LoDa. Similarly, when the image becomes blurry with dominant low-frequency components, our method still performs better. This is because the proposed frequency selection module (FSM) processes low-frequency and high-frequency features separately, enabling it to handle such situations effectively.

#### 4.7.4. Effect of Hand-Crafted Features

Table 8 presents an evaluation of the performance of the power grid dataset with different hand-crafted features: noise, brightness, and a combination of both. When only one type of hand-crafted feature is used, the performance decreases slightly. When no hand-crafted features are used, the performance drops significantly. When both are combined, the model achieves the best performance. These results indicate that noise and brightness features are crucial to improve performance and that hand-crafted features are indispensable.

#### 4.7.5. Effect of CNN Features

**Figure 8:**
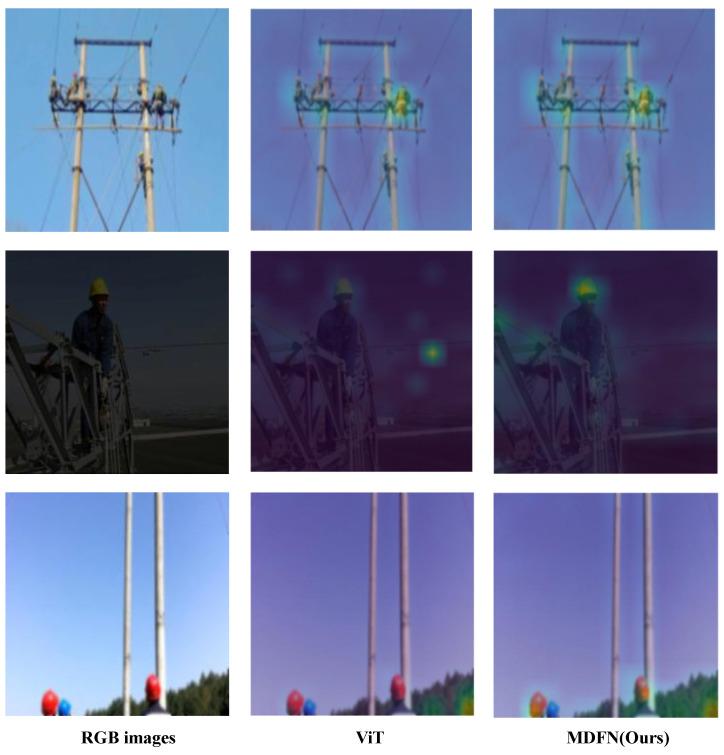
Visualization of attention maps of the CLS token towards the image features of ViT and the proposed MDFN.

To better illustrate the impact of integrating CNN features, the attention maps of the CLS token output by ViT are visualized, as shown in Figure 8. Compared to the original ViT, our method demonstrates a stronger response to targets in the images. In the first row, for example, the original ViT shows a weaker response to the utility pole, whereas our method provides a much stronger focus. In the second row, ViT fails to respond to the safety helmet, but our method effectively captures it. These results visually highlight the effectiveness of incorporating CNN features into ViT.

## 5. Conclusions

We propose a multi-dimension distortion feature network (MDFN) combining CNN and Transformer architectures to enhance image quality prediction by leveraging low- and high-frequency, noise, and brightness features. Two branches for feature extraction are used to obtain the local distortion representations and global image representations, respectively. The CNN branch is responsible for extracting the multi-scale distortion representation and the Transformer branch combines the features from CNN to achieve the fusion of the local and global features. In the CNN branch, the frequency selection module is proposed for obtaining more detailed distortion tokens, which helps the network obtain more accurate predictions. In addition, whereas previous methods solely use the CLS token for the prediction, we design an effective method to extract the noise and brightness features and combine them with the CLS token to obtain the prediction score. According to the results of our experiments, the performance of MDFN in image quality assessment is effectively improved, surpassing previous methods and the baseline method. Our novel and effective ideas, namely, that splitting the features into low- and high-frequency representations is more beneficial for predicting the quality score and that combining the noise and brightness representations can improve performance, are validated by these results.

## Figures and Tables

**Table 1:** Performance of different methods on the LIVE dataset, where the top two results are highlighted in bold. PLCC and SRCC are calculated by taking the median of the results from 10 runs.

Method	SRCC	PLCC
ILNIQE [41]	0.902	0.906
BRISQUE [42]	0.929	0.944
WaDIQaM-NR [43]	0.960	0.955
DB-CNN [44]	0.968	0.971
TIQA [21]	0.949	0.965
MetaIQA [19]	0.960	0.959
P2P-BM [45]	0.959	0.958
HyperIQA [8]	0.962	0.966
MUSIQ [40]	0.940	0.911
TReS [22]	0.969	0.968
LIQE [46]	0.970	0.951
DEIQT [10]	**0.979**	**0.981**
LoDa [11]	0.975	0.979
MDFN (Ours)	**0.981**	**0.983**

**Table 2:** Performance of different methods on the LIVEC dataset, where the top two results are highlighted in bold. PLCC and SRCC are calculated by taking the median of the results from 10 runs.

Method	SRCC	PLCC
ILNIQE [41]	0.508	0.508
BRISQUE [42]	0.629	0.629
WaDIQaM-NR [43]	0.682	0.671
DB-CNN [44]	0.851	0.869
TIQA [21]	0.845	0.861
MetaIQA [19]	0.835	0.802
P2P-BM [45]	0.844	0.842
HyperIQA [8]	0.859	0.882
MUSIQ [40]	0.702	0.746
TReS [22]	0.846	0.877
DEIQT [10]	0.875	0.894
LoDa [11]	**0.876**	**0.899**
MDFN (Ours)	**0.889**	**0.903**

**Table 3:** Performance of different methods on the TID2013 dataset, where the top two results are highlighted in bold. PLCC and SRCC are calculated by taking the median of the results from 10 runs.

Method	SRCC	PLCC
ILNIQE [41]	0.521	0.648
BRISQUE [42]	0.626	0.571
WaDIQaM-NR [43]	0.835	0.855
DB-CNN [44]	0.816	0.865
TIQA [21]	0.846	0.858
MetaIQA [19]	0.856	0.868
P2P-BM [45]	0.862	0.856
HyperIQA [8]	0.840	0.858
MUSIQ [40]	0.773	0.815
TReS [22]	0.863	0.883
DEIQT [10]	**0.892**	**0.908**
LoDa [11]	0.869	0.901
MDFN (Ours)	**0.880**	**0.909**

**Table 4:** Performance of different methods on the power grid dataset, where the top two results are highlighted in bold. PLCC and SRCC are calculated by taking the median of the results from 10 runs.

Method	SRCC	PLCC
DB-CNN [44]	0.973	0.980
TIQA [21]	0.971	0.975
HyperIQA [8]	0.973	0.977
DEIQT [10]	0.971	0.974
LoDa [11]	**0.976**	**0.982**
MDFN (Ours)	**0.981**	**0.989**

**Table 5:** Ablation experiments on the power grid dataset. The best result is highlighted in bold. HC denotes the hand-crafted features.

FSM	LDFM	HC	PLCC	SRCC
		✓	0.698	0.694
	✓		0.979	0.973
	✓	✓	0.981	0.975
✓	✓		0.981	0.975
✓	✓	✓	**0.989**	**0.981**

**Table 6:** Comparison of performance with different CNN backbones on the power grid dataset. The best result is highlighted in bold.

Backbone	PLCC	SRCC
ResNet18	0.978	0.974
VGG16	0.979	0.975
ResNet50	**0.989**	**0.981**

**Table 7:** Analysis of the frequency components on the power grid dataset. The best result is highlighted in bold.

Low-Frequency	High-Frequency	PLCC	SRCC
✓		0.979	0.975
	✓	0.981	0.976
✓	✓	**0.989**	**0.981**

**Table 8:** Comparison of perfomance with different hand-crafted features on the power grid dataset. The best result is highlighted in bold.

Hand-Crafted Features	PLCC	SRCC
None	0.981	0.975
Only Noise	0.987	0.980
Only Brightness	0.988	0.979
Both	**0.989**	**0.981**

## Data Availability

The original contributions presented in this study are included in the article; further inquiries can be directed to the corresponding author.

## Abbreviations

The following abbreviations are used in this manuscript:

AI	Artificial intelligence
ViT	Vision Transformer
CNN	Convolutional neural network
IQA	Image quality assessment
